# Hyperammonaemia in classic organic acidaemias: a review of the literature and two case histories

**DOI:** 10.1186/s13023-018-0963-7

**Published:** 2018-12-06

**Authors:** Johannes Häberle, Anupam Chakrapani, Nicholas Ah Mew, Nicola Longo

**Affiliations:** 1 Division of Metabolism and Children’s Research Centre, University Children’s Hospital Zurich, Zurich, Switzerland; 2 Department of Clinical Inherited Metabolic Disorders, Great Ormond Street Hospital for Children NHS Foundation Trust, London, UK; 3 Children’s National Rare Disease Institute, Children’s National Health System, Washington, DC, USA; 4 Department of Pediatrics, Division of Medical Genetics, University of Utah School of Medicine, 30 N 1900 E, Salt Lake City, UT 84132 USA

**Keywords:** Metabolic acidosis, Hyperammonaemia, Organic acidaemias, Metabolic decompensation, Biochemical pathogenesis

## Abstract

**Background:**

The ‘classic’ organic acidaemias (OAs) (propionic, methylmalonic and isovaleric) typically present in neonates or infants as acute metabolic decompensation with encephalopathy. This is frequently accompanied by severe hyperammonaemia and constitutes a metabolic emergency, as increased ammonia levels and accumulating toxic metabolites are associated with life-threatening neurological complications. Repeated and frequent episodes of hyperammonaemia (alongside metabolic decompensations) can result in impaired growth and intellectual disability, the severity of which increase with longer duration of hyperammonaemia. Due to the urgency required, diagnostic evaluation and initial management of patients with suspected OAs should proceed simultaneously. Paediatricians, who do not have specialist knowledge of metabolic disorders, have the challenging task of facilitating a timely diagnosis and treatment. This article outlines how the underlying pathophysiology and biochemistry of the organic acidaemias are closely linked to their clinical presentation and management, and provides practical advice for decision-making during early, acute hyperammonaemia and metabolic decompensation in neonates and infants with organic acidaemias.

**Clinical management:**

The acute management of hyperammonaemia in organic acidaemias requires administration of intravenous calories as glucose and lipids to promote anabolism, carnitine to promote urinary excretion of urinary organic acid esters, and correction of metabolic acidosis with the substitution of bicarbonate for chloride in intravenous fluids. It may also include the administration of ammonia scavengers such as sodium benzoate or sodium phenylbutyrate. Treatment with N-carbamyl-L-glutamate can rapidly normalise ammonia levels by stimulating the first step of the urea cycle.

**Conclusions:**

Our understanding of optimal treatment strategies for organic acidaemias is still evolving. Timely diagnosis is essential and best achieved by the early identification of hyperammonaemia and metabolic acidosis. Correcting metabolic imbalance and hyperammonaemia are critical to prevent brain damage in affected patients.

## Background

Organic acidaemias (OAs), a group of rare, inherited, metabolic disorders, typically present in neonates or infants as acute, potentially life-threatening, metabolic decompensation [1–3], which follows an initial symptom-free period of a few days [3, 4]. An increase in plasma ammonia to toxic levels is often a key feature of metabolic decompensation [5, 6], which needs to be addressed promptly since it can lead to life-threatening neurological complications [7]. Paediatricians, who do not have specialist knowledge of metabolic disorders, have the challenging task of initiating treatment while obtaining a timely diagnosis. This article provides practical advice to help with the decision-making and clinical management of hyperammonaemia during early, acute metabolic decompensation in neonates and infants with OAs.

Although more than 65 different OAs have been described, even the more common ones are rarely encountered by paediatricians, as the collective incidence is about 1 in 3000 live births [8]. The initial signs and symptoms of metabolic decompensation – such as poor feeding, vomiting, dehydration and lethargy – are non-specific and can mimic other conditions, such as bacterial sepsis [3], thereby making rapid identification and intervention to prevent irreversible neurological sequelae [3, 4] particularly challenging. Elevated plasma ammonia levels have a direct impact on neurological outcomes [3, 9], with levels above 200 μmol/L usually being associated with drowsiness and lethargy (impaired vigilance) [5].

This article describes the three OAs most likely to result in hyperammonaemia during acute metabolic decompensation: propionic acidaemia (PA; MIM# 606054), methylmalonic acidaemia (MMA; MIM# 251000) and isovaleric acidaemia (IVA; MIM# 243500). Together with maple syrup urine disease, PA, MMA and IVA are sometimes referred to as ‘classic’ OAs [10]. Other OAs cause typical biochemical changes identified through newborn screening and have different clinical implications. While hyperammonaemia is an important manifestation of PA, MMA and IVA, its clinical significance needs to be considered together with other abnormalities, such as acidosis, ketosis, and lactic acidaemia, as well as distinguishing it from the hyperammonaemia caused by urea cycle disorders (UCDs) [11], the main group of conditions from which OAs must be differentiated [3, 12, 13]. Not all patients with PA, MMA or IVA present with hyperammonaemia, but most develop it in the newborn period, and continue to have recurrent episodes of hyperammonaemia during metabolic decompensation [3, 14].

This article considers the pathophysiology and underlying biochemical pathogenesis of OAs, and the diagnostic and clinical management of hyperammonaemia in PA, MMA and IVA. Two case studies illustrate the clinical presentation, key diagnostic evaluations and treatment in a neonate with acute decompensation and in a child with chronically elevated ammonia levels.

## Clinical presentation of OAs

The development of an acute, life-threatening illness in a previously apparently healthy neonate is a strong indicator of an inherited metabolic disorder [15]. At initial presentation of neonates, the normal diet of breast milk or formula, together with postpartum catabolism, causes acute decompensation. In patients with OAs beyond the neonatal period, acute attacks are usually triggered by conditions that increase catabolism, such as fasting, vomiting or febrile infections (Table 1) [5, 13, 16].

**Table 1:** Triggers, clinical signs and symptoms, and biochemical signs of acute decompensation in PA and MMA [3]

Triggers	Clinical signs and symptoms	Biochemical signs
Infection	Poor feeding	Metabolic acidosis: • pH < 7.3 • anion gap > 20 mmol/L • low HCO_3_^−^ or base excess <−5 mmol/L)
Fever	Vomiting	
Prolonged fasting	Lethargy	
Medication (e.g. chemotherapy, high-dose glucocorticoids)	Seizures	
Surgery and/or general anaesthesia	Irritability	Elevated blood lactate (> 3 mmol/L)
Acute trauma	Respiratory distress	Hyperammonaemia: • > 75 μmol/L associated with symptomatic decompensation • > 200 μmol/L associated with impaired vigilance [5]
Significant haemorrhage	Hypothermia	
Psychological stress	Dehydration	
Excessive protein intake	Weight loss	
Excessive physical exertion		Ketonuria (> trace in infants or > + in children)
		Neutropenia
		Thrombocytopenia

Abbreviation: *HCO*_*3*_^*−*^ Bicarbonate, *MMA* Methylmalonic acidaemia, *PA* Propionic acidaemia

In the newborn period, poor feeding, vomiting, dehydration, lethargy, tachycardia and hypothermia (Table 1) usually develop within 2–7 days after birth [3, 13] and, if left untreated, can quickly progress to respiratory distress, coma and death [16, 17]. However, as these symptoms are also observed in more common conditions, such as infection, a high index of suspicion should be maintained [18]. If there is neurological involvement [3, 19], routine laboratory studies for OAs (chemistry profile, ammonia level) should be performed at the same time as the septic work-up.

After the neonatal period, OAs may present with delays in development, failure to thrive, altered mental state, abnormal tone, seizures and motor dysfunction [16, 20, 21]. Other signs and symptoms of acute or chronic decompensation can include weight loss (often seen in newborns) and bone-marrow suppression in PA and MMA, and the characteristic ‘sweaty feet’ odour in patients with IVA [16, 22–24].

## Biochemical pathogenesis of OAs

**Fig. 1:**
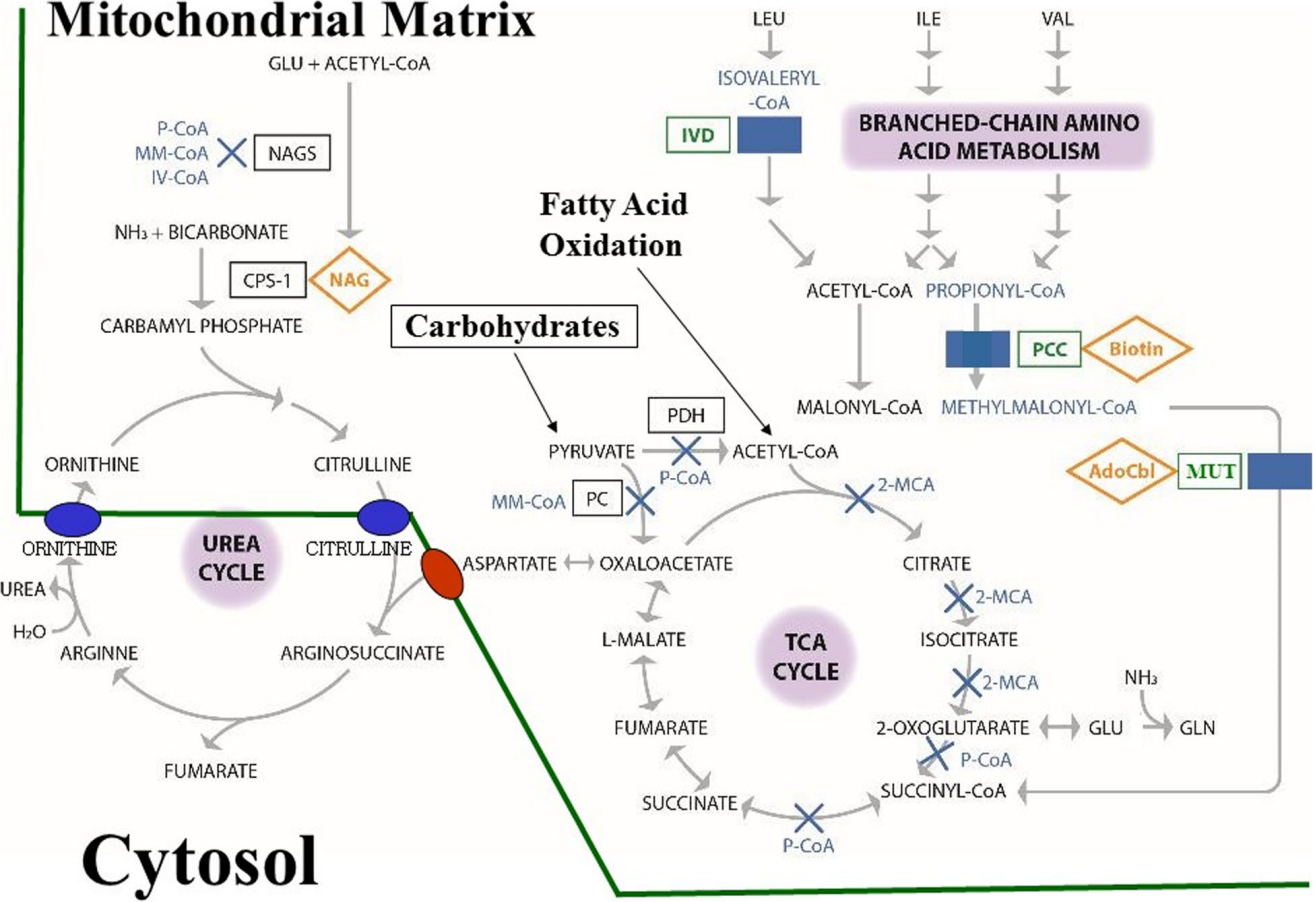
Proposed biochemical pathogenesis of organic acidaemias: propionic acidaemia, methylmalonic acidaemia, isovaleric acidaemia. Genetic defects in enzymes involved in the breakdown of amino acids cause the accumulation of toxic organic acids with disruption of the tricarboxylic acid and urea cycles. Propionic acidaemia is caused by propionyl-CoA carboxylase deficiency, and methylmalonic acidaemia results from methylmalonyl-CoA mutase deficiency [16, 17]. Isovaleric acidaemia is caused by isovaleryl-CoA dehydrogenase deficiency, which is involved in leucine catabolism. Rectangles indicate key affected enzymes: green rectangles indicate the primary affected enzymes (propionyl-CoA carboxylase, methylmalonyl-CoA mutase, isovaleryl-CoA dehydrogenase); blue solid rectangles are positions of primary enzyme blocks. Blue crosses indicate secondary enzyme inhibition; blue texts are enzyme precursors; orange diamonds are key enzyme co-factors. Abbreviations: *2-MCA* 2-methylcitrate, *CoA* coenzyme A, *CPS-1* carbamyl phosphate synthetase-1, *GLN* glutamine, *GLU* glutamate, *H*_*2*_*O* water, *IV-CoA* isovaleryl-CoA, *IVD* isovaleryl-CoA dehydrogenase, *LEU* leucine, *MM-CoA* methylmalonyl-CoA, *MUT* methylmalonyl-CoA mutase, *NAG* N-acetylglutamate, *NAGS* N-acetylglutamate synthase, *NH*_*3*_ ammonia, *PC* pyruvate carboxylase, *PCC* propionyl-CoA carboxylase, *P-CoA* propionyl-CoA, *PDH* pyruvate dehydrogenase complex, *TCA* tricarboxylic acid, *VAL* valine. Modified from Kölker et al. 2013 [2], Schiff et al. 2016 [17], and Vianey-Saban et al. 2006 [29]

All the classical OAs are inherited as autosomal recessive traits [17] and are caused by a deficiency in the enzymes involved in breaking down amino acids (isoleucine, valine, methionine and threonine in PA and MMA, and leucine in IVA) and additional substrates (odd-chain fatty acids, cholesterol, nucleotides in PA and MMA) [14, 16, 17, 25, 26] (Fig. 1). PA is caused by a deficiency in propionyl-coenzyme A (CoA) carboxylase, composed of alpha and beta subunits encoded by the *PCCA* and *PCCB* genes, respectively [17]. MMA can be caused by a deficiency of multiple enzymes affecting either the transformation of cobalamin to adenosylcobalamin, or the activity of methylmalonyl-CoA mutase, or racemase [16, 17, 27, 28]. IVA is caused by a deficiency in isovaleryl-CoA dehydrogenase [16, 17]. Each enzyme deficiency leads to a build-up of its precursor(s): propionyl-CoA (P-CoA) in PA, P-CoA and methylmalonyl-CoA in MMA, and isovaleryl-CoA in IVA [16, 29], and the corresponding organic acids once released from CoA (Fig. 1).

Hyperammonaemia likely results from the interaction of the substrates for the defective enzymes with other biochemical pathways, including the urea and tricarboxylic acid (TCA) or Krebs cycles (Fig. 1) [2, 17, 29]. In the urea cycle, a series of enzymes and transporters facilitate the incorporation of ammonia (as the amino group of carbamyl phosphate and aspartate) into urea for urinary excretion (Fig. 1). The complete urea cycle is mostly active in the liver, where, under physiological conditions, ammonia is removed in the mitochondrial matrix and cytosol of periportal hepatocytes [30]. N-acetylglutamate synthase (NAGS; EC 2.3.1.1) catalyses the formation of N-acetylglutamate (NAG) from glutamate and acetyl-CoA; NAG is an essential activator for the first enzyme in the urea cycle (carbamyl phosphate synthetase 1; CPS-1). Ammonia that escapes the urea cycle in periportal hepatocytes conjugates with glutamate to form glutamine in pericentral hepatocytes, explaining the increase in glutamine seen in all UCDs [30]. The urea cycle interacts with other mitochondrial pathways such as the TCA cycle, fatty acid oxidation and amino acid catabolism (Fig. 1) [2, 17, 29]. The TCA cycle enables the extraction of reducing equivalents from acetyl-CoA that fuel the mitochondrial electron transport chain to generate adenosine triphosphate through oxidative phosphorylation. The TCA and urea cycles are linked at oxaloacetate, the start and end point of the TCA cycle (Fig. 1), where oxaloacetate can be converted to aspartate via transamination [2, 29]. In addition, glutamate can be generated from 2-oxoglutarate and ammonia during physiological cataplerosis [2, 17, 29]. The oxidation of fatty acids, carbohydrates and amino acids generates acetyl-CoA, one of the substrates necessary for NAG synthesis [31, 32].

In OAs, the accumulated metabolites (P-CoA, methylmalonyl-CoA and isovaleryl-CoA) compete with acetyl-CoA to inhibit the activity of NAGS [12, 33, 34], thereby diminishing the synthesis of carbamyl phosphate [35]. This secondary impairment of the urea cycle can lead to elevated ammonia levels with neurotoxic effects during metabolic decompensation in OAs [12, 29, 36]. In contrast, the hyperammonaemia observed in UCDs is caused by primary defects in urea cycle enzymes or transporters [32].

The end point of propionic and methylmalonic acid metabolism is the generation of succinyl-CoA, an important intermediate of the TCA cycle [16]. This degradation pathway (anaplerosis) represents an important mechanism for replenishing TCA cycle intermediates [13]. Since anaplerosis is disrupted in PA and MMA, TCA cycle intermediates must be derived from other pathways. For instance, glutamine can be cleaved to form ammonia and glutamate; this in turn splits into ammonia and 2-oxoglutarate, which can then enter the TCA cycle (Fig. 1) [13]. A by-product of these reactions is the release of ammonia, which may contribute to the chronic hyperammonaemia observed in these disorders. In addition, the reduced concentration of glutamate might further impair synthesis of NAG by NAGS. Finally, the flow of glutamine to α-ketoglutarate to replenish the TCA cycle results in a decrease, rather than an increase, in glutamine levels in PA as ammonia levels increase [13].

Hyperammonaemia toxicity in the developing brain is due to multifactorial mechanisms, including disruption of amino acid and cerebral energy metabolism, and increased oxidative stress [36]. Excess propionic and methylmalonic acid levels also appear to contribute directly to progressive neurological deterioration by acting through protein kinases and phosphatases to interfere with cytoskeletal assembly in neuronal and glial cells [8], as well as disrupting the mitogen-activated protein kinase and p53 signalling pathways that promote apoptosis of neuronal cells [8, 37].

In addition to propionic acid and methylmalonic acid, other toxic metabolites such as propionylcarnitine and methylcitric acid can also disrupt the normal functioning of the TCA cycle [8, 12]. There is increasing evidence to suggest that the significant disruption of mitochondrial function – involving multiple deficiencies in oxidative phosphorylation via the mitochondrial electron transport chain, secondary to inhibition of the TCA cycle [25, 38] – culminates in a synergistic impairment of energy metabolism and depletion of cellular energy stores [2, 29]. Elevated levels of propionic acid, methylmalonic acid and isovaleric acid appear to directly mediate oxidative stress by increasing levels of reactive oxygen species (ROS) [8], and reducing ROS-protective levels of glutathione [39]. This further contributes to the intra- and extracerebral disease manifestations of the classical OAs [2]. Mitochondrial electron transport chain dysfunction has been linked to damage in the basal ganglia [1, 19, 25]; it has been suggested that methylcitric acid may be more neurotoxic than methylmalonic acid [40]. In addition, intracellular metabolite accumulation leads to CoA sequestration and its subsequent depletion, resulting in detrimental effects primarily in mitochondria [41].

## Clinical consequences and complications

Developmental delays are common in OAs, with some patients showing progressive neurological deterioration with increasing age. In a retrospective study of 55 patients with PA (median age 5.2 years), 75.5% showed some degree of intellectual disability, with a median IQ of 55 [42]. Another study in 80 patients with classical OAs, found that PA was associated with the worst neurological prognosis: 37% of patients had abnormal neurological findings, 61% had abnormal psychometric scores, and 56% had basal ganglia lesions. In contrast, all patients with IVA had normal neurological examinations, only 18% had psychometric impairment, and only 17% had basal ganglia lesions [43].

OAs are also associated with long-term systemic effects in other organs with high energy requirements, such as the heart, kidneys, eyes [12, 25, 44, 45], pancreas [22] and bone marrow [23]. These extracerebral complications are apparently related to the synergistic effects of toxic metabolites and impaired mitochondrial oxidative phosphorylation [2, 25], often occur despite good metabolic control, and can appear in asymptomatic individuals [2, 12, 42, 46]. This may be related to sequestration of CoA within mitochondria, depletion of mitochondrial DNA, increased production of ROS, and alterations in gene expression [2]. Dysfunction of the mitochondrial electron transport chain may contribute to late multi-organ complications in PA and MMA [25], including the formation of megamitochondria in kidney proximal tubules, seen in MMA patients with chronic kidney disease [38]. For patients with PA, the depletion of carnitine and the sequestration of CoA by excess organic acids are further responsible for decreases in fatty acid oxidation and acetyl-CoA production [29, 41], in turn leading to further secondary inhibition of the urea and TCA cycles [29], and contributing to long-term cardiomyopathy [12].

## Clinical management and decision-making in OAs

Table 2 provides a clinical checklist aimed at paediatricians who are unfamiliar with OAs. This checklist complements current guidance [3] and outlines symptoms that may relate to an underlying diagnosis, laboratory testing requirements, clinical laboratory findings (Table 1), and immediate management recommendations for patients with OAs.

**Table 2:** Clinical checklist: acute management of organic acidaemia (modified from Baumgartner et al. 2014 [3])

• Acute management is required any time a patient with an organic acidaemia has symptoms such as lethargy, vomiting, tachypnoea, and impaired vigilance.	
• Laboratory testing (metabolic panel with ammonia, electrolytes, anion gap, lactate, ketone bodies in urine) can determine whether the patient needs urgent care. Plasma amino acids, urine organic acids and plasma acylcarnitine profile are useful to establish the cause of acute metabolic decompensation and to monitor long-term patient management.	
• Immediate administration of sufficient calories (100–120 kcal/kg/day in infants, with lower amounts in older children) in the form of glucose and lipids is necessary during acute decompensation in organic acidaemias, although protein should be restarted as soon as possible (usually not later than 24–48 h). As the patient improves, a nasogastric tube should be inserted to administer enteral formulas containing limited amounts of proteins (0.5 g/kg/day). Enteral feeds should be gradually increased to provide adequate calories (100–120 kcal/kg/day in infants, with lower requirements in older children) and protein (increasing natural protein to 0.8–1.2 g/kg/day and then adding the balance of protein needed via medical foods without propionic acid precursors to reach the recommended daily allowance for age).	
• Intravenous glucose may be given as 10% dextrose (D10), or 20% dextrose (D20) if a central line is available, together with appropriate salts (half-normal saline up to about 5 years of age, normal saline after 5 years of age; potassium chloride at 20 mEq/L if there is no evidence of hyperkalaemia), and intralipids 20% to provide adequate calories for age.	
• In case of metabolic acidosis (sodium bicarbonate < 15 mEq/L), sodium bicarbonate is substituted for sodium chloride (75 mEq/L or 150 mEq/L), and potassium acetate (20 mEq/L) is substituted for potassium chloride. Serum bicarbonate and electrolytes should be monitored every 4–6 h and intravenous sodium bicarbonate should be switched to sodium chloride once serum bicarbonate reaches 25 mEq/L.	
• If glucose becomes greater than 8.3 mmol/L, insulin should be given: 0.1 U/kg as a bolus, followed by 0.1 U/kg/hour as a drip. Insulin dose should be adjusted to maintain glucose levels 3.9–8.3 mmol/L.	

### Diagnosis

To prevent the risk of neurological sequelae, treatment of OAs should be initiated as soon as the condition is suspected. Advice from a metabolic specialist should be sought quickly to allow simultaneous diagnostic work-up and initial management [3]. Routine clinical laboratory investigations often show metabolic acidosis, increased anion gap, hyperammonaemia, ketosis, and lactic acidosis (Table 1) [3, 15, 47]. OAs are diagnosed biochemically by analysing the levels of organic acids in urine, with additional information derived from the plasma acylcarnitine and amino acid profiles (the latter to exclude UCDs); acylcarnitine results may be the first available. In urine, significant elevations of methylcitric acid in the absence of elevated MMA is diagnostic of PA [3]. In MMA, methylmalonic acid is markedly increased, and methylcitric acid may be minimally elevated. Elevated concentrations of 3-hydroxyisovaleric acid and isovalerylglycine are characteristic of IVA [48, 49]. The duration of hyperammonaemia, abnormal acid-base balance with metabolic acidosis (decreased pH and bicarbonate with increased anion gap), and the duration of coma correlate with poor neurological outcomes. In a sick infant, these abnormalities should be corrected as rapidly as possible.

OAs must be differentiated from UCDs. In both cases, patients usually present with neurological symptoms and hyperammonaemia [50]. The presence of metabolic acidosis and ketonuria suggests OA in neonates (Table 3), whereas respiratory alkalosis is often seen in UCDs [15]. Plasma amino acids can pinpoint specific abnormalities in the urea cycle. Glutamine is typically high in UCDs and can be low to normal in OAs; glycine may be elevated in both conditions [3, 50, 51]. However, it is important to exclude non-ketotic hyperglycinaemia, which can also cause encephalopathy in infants [52]. Urine orotic acid, measured as part of the urine organic acids profile or by separate analysis, is elevated in the most common UCD (X-linked ornithine transcarbamylase deficiency), but is normal or only minimally elevated in NAGS or CPS-1 deficiencies and in all OAs [32, 53].

**Table 3:** Clinical laboratory findings in patients with PA, MMA, or UCDs (modified from Baumgartner et al. 2014 [3])

	PA/MMA	UCDs
Hyperammonaemia	+ to ++	++
Acidosis	+	+/−
Ketonuria^a^	++/+++	–
Hypoglycaemia	+/−	–
Increased lactate^b^	+	–
Increased AST and ALT	+/−	+
Increased uric acid	+	–
Decreased blood cell counts	+	–

Abbreviations: *ALT* Alanine transaminase, *AST* Aspartate transaminase, *MMA* Methylmalonic acidaemia, *PA* Propionic acidaemia, *UCDs* Urea cycle disorders

^a^Ketonuria ++/+++ suggests OA in neonates

^b^Plasma lactate > 6 mmol/L (levels of 2–6 mmol/L may be due to severe crying or extensive muscle activity)

### Acute management

Current guidelines for managing OAs focus on PA and MMA [3]. The initial aim of therapy is to reverse endogenous catabolism and provide sufficient energy to promote anabolism [3]. Sufficient calories should be provided by intravenous glucose and lipids to prevent further catabolism, and metabolic acidosis can be corrected by substituting bicarbonate and acetate for chloride in intravenous (IV) solutions. Infants require 100–120 kcal/kg/day, with lower requirements in older children. Insulin should be used to correct the hyperglycaemia caused by the administration of IV glucose and to reverse catabolism; it is important to monitor serum/plasma phosphate, magnesium, calcium and potassium during administration of IV insulin and to promptly correct electrolytic imbalance.

As amino acids are the primary precursors of organic acids, protein intake should be transiently stopped (maximum 24–48 h) [3]. Carnitine (recommended dosage of 200 mg/kg/day) should be given to promote the urinary excretion of organic acid-carnitine esters. As the patient improves, a nasogastric tube should be inserted to administer enteral formulas containing limited amounts of proteins (0.5 g/kg/day). Enteral feeds should be gradually increased to provide adequate calories (100–120 kcal/kg/day in infants, with lower requirements in older children) and protein (increasing natural protein to 0.8–1.2 g/kg/day and then adding the balance of protein needed via medical foods without propionic acid precursors to reach the recommended daily allowance for age) [3].

There are currently no specific guidelines for managing IVA. In general, the management approach for this OA is similar to that for PA or MMA except that leucine restriction and glycine supplementation are introduced to reduce the accumulation of isovaleric acid and promote its excretion [54, 55].

Sodium benzoate or sodium phenylbutyrate are the mainstay of ammonia detoxification in patients with UCDs. These medications provide an alternative pathway for nitrogen excretion through CoA-dependent enzymatic reactions. Sodium benzoate binds to glycine to form hippuric acid, and sodium phenylbutyrate is first metabolised to phenylacetate, which binds to glutamine to form phenylacetylglutamine. Both hippuric acid and phenylacetylglutamine are excreted in the urine. Sodium benzoate is also used sometimes in patients with PA and MMA. For patients with undiagnosed symptomatic hyperammonaemia (> 150–< 250 μmol/L for neonates; 100–250 μmol/L for non-neonates) [3], current guidance recommends the use of sodium benzoate or sodium phenylbutyrate [3, 7, 12]. Unlike UCDs, these therapies should be used with caution in patients with OAs, because glutamine levels can already be low due to the abnormal functioning of the TCA cycle in PA and MMA [3, 12].

Treatment with N-carbamyl-L-glutamate (NCG; Carbaglu®, Orphan Europe), a stable synthetic analogue of NAG, the essential co-factor of CPS-1, can stimulate the first step of the urea cycle (Fig. 1), enabling the formation of carbamyl phosphate and reducing ammonia levels in patients with secondary hyperammonaemia [6, 56, 57]. Two retrospective observational studies [35, 58] and case reports [56, 59, 60] indicate that NCG can reduce ammonia levels in neonates and older patients with PA, MMA or IVA, irrespective of the use of scavenger medication [6]. NCG is recommended for the management of patients with undiagnosed symptomatic hyperammonaemia, and is also included in the 2014 European guidance for managing PA and MMA [3].

Haemodialysis can be used in severely encephalopathic patients with hyperammonaemia who are not responding to medical management [35].

### Long-term management

The aims of long-term OA management are to prevent episodes of metabolic decompensation, minimise complications, and promote normal growth and development [3, 61]. This involves a threefold approach: dietary management; drug therapy; and life-long monitoring to identify and treat potential complications, such as pancreatitis, renal impairment, cardiomyopathy, cardiac arrhythmias and osteoporosis [12].

Low-protein diets are an essential part of long-term management for all classical OAs [61, 62]. Patients with PA or MMA should also receive supplementation with precursor-free synthetic amino acid mixtures, vitamins, minerals and fats [61, 63]. In general, infants and young children require 0.8–1.2 g/kg/day of natural protein, with the balance of protein requirement coming from medical foods not containing propionic acid precursors, in order to achieve the recommended daily allowance appropriate for age. After 2 years of age, some children with methylmalonic acidaemia can continue with protein restriction alone, with the balance of calories provided by protein-free foods.

The most commonly used treatments are L-carnitine, vitamin B12 (as hydroxocobalamin in cobalamin-responsive MMA), and intermittent antibiotics (e.g. metronidazole) to reduce intestinal flora producing propionic acid [3]. Citrate supplementation (7.5 mEq/kg/day), which can buffer metabolic acidosis and replenish the TCA cycle, may also be beneficial in PA [64]. In patients with IVA, long-term management also typically involves glycine and carnitine supplementation in addition to a low-protein diet [54, 55, 65, 66]. Patients with OAs in good metabolic control, intended as the adequate provision of calories with limited amounts of protein and the supplements listed above, are usually clinically stable (although with mildly elevated ammonia levels in some cases). Metabolic control in many cases requires the use of a gastrotomy tube, which facilitates the administration of calories and of feeds at night to prevent catabolism. This precarious equilibrium can be altered by persistent vomiting or intercurrent infections, which can decrease calorie intake and increase calorific needs, leading to hyperammonaemia and, in some cases, metabolic acidosis.

NCG may also be effective in the long-term treatment in patients with severe PA and MMA with recurrent episodes of hyperammonaemia [67]. In a recent study, eight patients with PA or MMA who had experienced between three and 11 episodes of decompensation in the preceding year were treated with NCG (50 mg/kg/day) for 7–16 months. Treatment with NCG markedly decreased the number and severity of decompensation episodes, with three patients experiencing no additional episodes during treatment, and with all episodes in the remaining patients becoming amenable to treatment at home [67].

In a separate published case report, one patient had experienced 78 admissions for decompensations during the first 9 years of life, with 7–10 admissions per year [68]. Continuous treatment with NCG was initiated at 9 years of age at 100 mg/kg/day with a dose reduction to 50 mg/kg/day after 6 months. In the period from 9 to 15 years of age, the patient experienced only two episodes of acute decompensation requiring hospitalisation, both of which occurred during the first year of treatment with NCG. While these findings provide some evidence for the long-term role of NCG in patients with severe PA and MMA who experience recurrent episodes of hyperammonaemia, there are no double-blind, placebo-controlled studies available. Consideration also needs to be given to reports of NCG being ineffective in OAs [57], indicating the need for more studies exploring the long-term effects of NCG on hyperammonaemia in OAs.

In patients with PA or MMA who experience repeated metabolic decompensation, or in whom the disease is difficult to manage with diet and pharmacological therapy, liver transplantation can reduce the number of hospitalisations and improve quality of life [3, 12]. Although liver transplantation may reduce the risk of certain complications, such as cardiomyopathy, it has no effect on the risk of neurological or ophthalmological complications, and, of course, has associated risks of mortality [3, 12]. For some adult patients with MMA-related end-stage kidney disease, renal transplantation is also indicated [12].

## Case studies

The following cases illustrate: (1) the diagnostic work-up and initial management of acute decompensation in a 2-day-old neonate; (2) the diagnostic work-up and initial and long-term management of chronically elevated blood ammonia in an infant aged 9 months at diagnosis.

### Case study 1: acute decompensation in the neonatal period

A 2-day-old girl presented with poor feeding, dehydration and lethargy progressing to coma. Initial laboratory investigations indicated severe metabolic acidosis and hyperammonaemia. The patient was intubated and given intravenous fluids containing glucose, sodium bicarbonate (75 mEq/L), potassium acetate (20 mEq/L), intralipids (20%) and insulin (starting with a 0.1 U/kg bolus, followed by 0.1 U/kg/hour) to maintain glucose levels at 3.9–8.3 mmol/L. The key to treatment is to provide sufficient calories (110–120 kcal/kg/day) with the above fluids without creating osmotic imbalances.

**Fig. 2:**
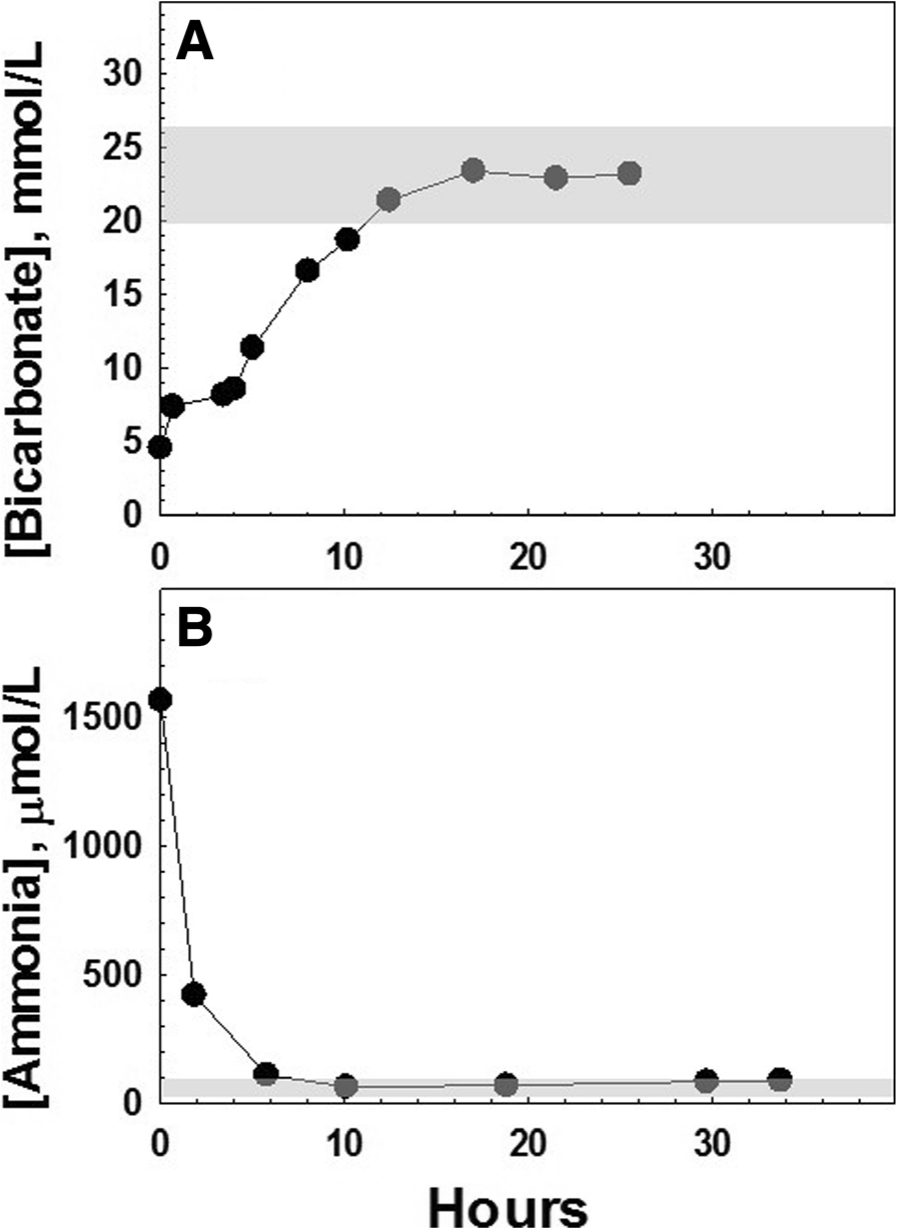
Normalisation of serum bicarbonate (**a**) and ammonia (**b**) after starting intravenous therapy in a 2-day-old neonate with acute decompensation of propionic acidaemia. Normal range is indicated by the shaded area

Ammonia levels normalised within 12 h and the patient was subsequently diagnosed with PA by urine organic acid analysis (3-OH-propionic acid = 5010 mmol/mol creatinine; methylcitric acid = 1982 mmol/mol creatinine). The plasma acylcarnitine profile indicated elevated propionylcarnitine (11.78 μmol/L; normal = < 0.55 μmol/L) with low levels of free carnitine (4 μmol/L; normal = 22–63 μmol/L). Figure 2 shows the progressive normalisation of serum bicarbonate (A) (normal range 20–26 mmol/L) and ammonia (B) (normal range 20–99 μmol/L in newborns) after starting intravenous therapy.

### Case study 2: chronically elevated blood ammonia in a young child

A 6.5-year-old boy was diagnosed with PA at 9 months of age during selective metabolic screening for intellectual disability. Ammonia was elevated (maximum 105 μmol/L) at diagnosis, but levels returned to the normal range (< 48 μmol/L) after corrective treatment (diet and carnitine supplementation at 100 mg/kg/d in two doses). Over the course of the following years, the patient had episodes of hyperammonaemia (maximum level: 378 μmol/L) that rapidly normalised after the administration of a single dose of sodium benzoate (200 mg/kg/dose given over 90 min).

For the majority of the measurements during the first year after diagnosis, ammonia levels taken during outpatient clinic visits were elevated in the range of 70–140 μmol/L), but there were no signs of acute hyperammonaemia and so no specific treatment was administered. These ammonia elevations were not considered to be causing a clinical problem, and were rather interpreted as an indication of suboptimal metabolic stability. Therefore, ammonia elevations were managed by optimising the patient’s diet and nutritional status.

**Fig. 3:**
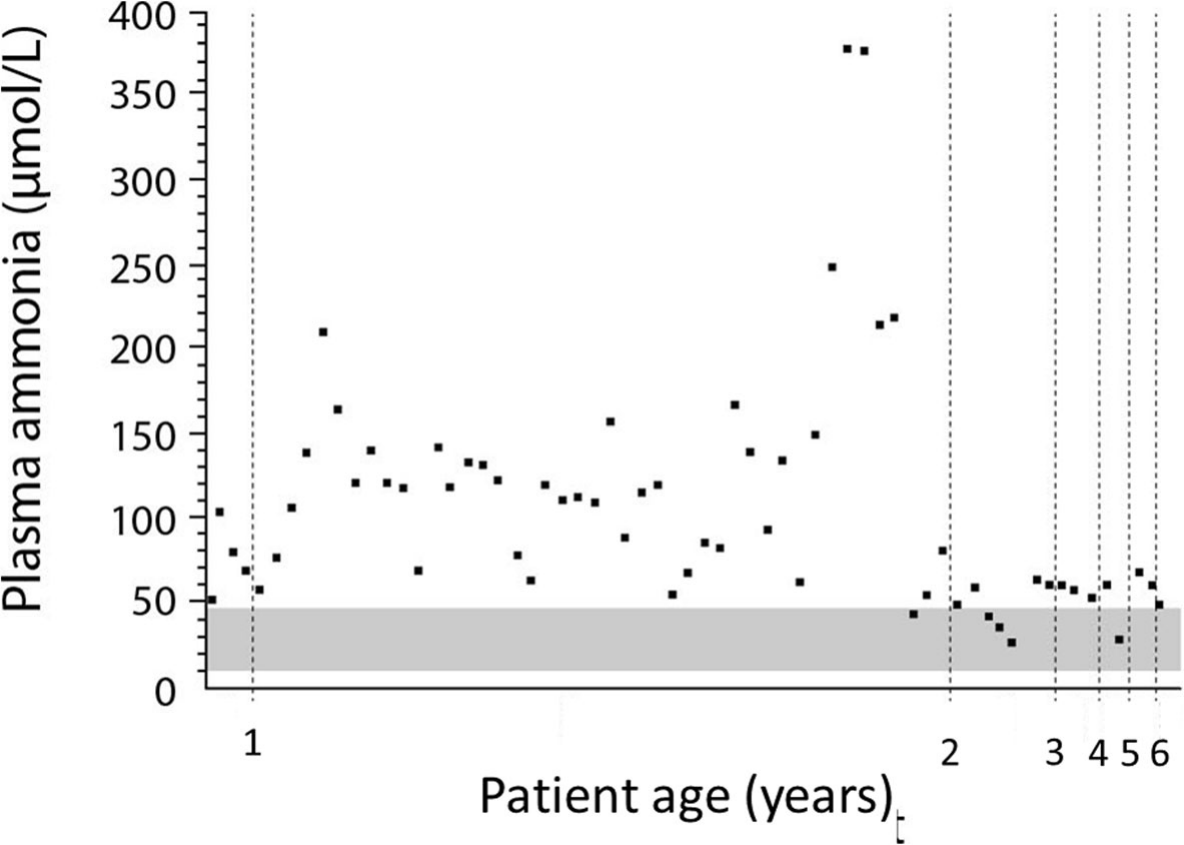
Plasma ammonia concentrations in a 6.5-year-old patient over a time period of 5.5 years after diagnosis was made at age 9 months. Concentrations are given in μmol/L and normal range is indicated by a shaded area

About 1 year after diagnosis, the patient received a gastrostomy tube. This facilitated dietary management to a large extent and, in particular, ensured a sufficient daily intake of calories. Once this was established, metabolic stability was much improved (Fig. 3). From diagnosis, the patient was given a low-protein diet, according to current guidelines [3]: initial natural protein intake was 0.9 g/kg/d but was gradually increased to 1.24 g/kg/d at age 5 years. Synthetic protein intake started at 0.8 g/kg/d and was gradually reduced to 0.24 g/kg/d at 6.5 years of age. This was done according to plasma amino acid monitoring and closely following the safe levels of protein recommendations in the Food and Agriculture Organization of the United Nations/World Health Organization/United Nations University expert consultation (2007) [69] (as detailed in [3]).

## Conclusions

Hyperammonaemia, frequently observed in the classical OAs (PA, MMA and IVA), is most often associated with metabolic decompensation in the neonatal period, and contributes to the risk of neurological damage. Timely diagnosis and intervention is essential, as prognosis is strongly linked to elevated blood ammonia levels and duration of coma, as well as to the increased levels of specific organic acids. Prompt evaluation from a metabolic specialist is required, but should not delay intervention, diagnostic work-up and initial management.

While the diagnosis of OAs remains challenging because of the low incidence of these disorders, as well as their variable and non-specific presentation, recent advances in diagnostic techniques, such as newborn screening by tandem mass spectrometry [11], which allow earlier identification, are encouraging. Screening of neonates for OAs and other inherited metabolic disorders offers the potential for early diagnosis and therapy [63]; however, newborn screening is not offered in all countries, and evidence that screening improves long-term outcomes is currently limited [1, 14, 50, 63, 69]. In many cases of OAs, results become available after newborns are already symptomatic.

With the diverse range of interventions in OAs, timely diagnosis by the identification of elevated ammonia levels and metabolic acidosis can allow their rapid correction, which is critical for the prevention of brain damage [46, 50].

## Abbreviations

2-MCA: 2-methylcitrate
ALT: Alanine transaminase
AST: Aspartate transaminase
CoA: Coenzyme A
CPS-1: Carbamyl phosphate synthetase-1
GLN: Glutamine
GLU: Glutamate
H_2_O: Water
HCO_3_: Bicarbonate
IV: Intravenous
IVA: Isovaleric acidaemia
IV-CoA: Isovaleryl-CoA
IVD: Isovaleryl-CoA dehydrogenase
Leu: Leucine
MMA: Methylmalonic acidaemia
MM-CoA: Methylmalonyl-CoA
MUT: Methylmalonyl-CoA mutase
NAG: N-acetylglutamate
NAGS: N-acetylglutamate synthase
NCG: N-carbamyl-L-glutamate
NH_3_: Ammonia
OA: Organic acidaemia
PA: Propionic acidaemia
PC: Pyruvate carboxylase
PCC: Propionyl-CoA carboxylase
P-CoA: Propionyl-CoA
PDH: Pyruvate dehydrogenase complex
ROS: Reactive oxygen species
TCA: Tricarboxylic acid
UCD: Urea cycle disorder
VAL: Valine

## References

[1] Dionisi-Vici C, Deodato F, Röschinger W, Rhead W, Wilcken B (2006). “Classical” organic acidurias, propionic aciduria, methylmalonic aciduria and isovaleric aciduria: long-term outcome and effects of expanded newborn screening using tandem mass spectrometry. J Inherit Metab Dis.

[2] Kölker S, Burgard P, Sauer SW, Okun JG (2013). Current concepts in organic acidurias: understanding intra- and extracerebral disease manifestation. J Inherit Metab Dis.

[3] Baumgartner MR, Hörster F, Dionisi-Vici C, Haliloglu G, Karall D, Chapman KA (2014). Proposed guidelines for the diagnosis and management of methylmalonic and propionic acidemia. Orphanet J Rare Dis.

[4] Ah Mew NA, Viall S, Kirmse B, Chapman KA (2015). Deconstructing black swans: an introductory approach to inherited metabolic disorders in the neonate. Adv Neonatal Care.

[5] Zwickler T, Haege G, Riderer A, Hörster F, Hoffmann GF, Burgard P (2012). Metabolic decompensation in methylmalonic aciduria: which biochemical parameters are discriminative?. J Inherit Metab Dis.

[6] Chapel-Crespo CC, Diaz GA, Oishi K (2016). Efficacy of N-carbamoyl-L-glutamic acid for the treatment of inherited metabolic disorders. Expert Rev Endocrinol Metab.

[7] Matoori S, Leroux J (2015). Recent advances in the treatment of hyperammonemia. Adv Drug Deliv Rev.

[8] Villani GRD, Gallo G, Scolamiero E, Salvatore F, Ruoppolo M (2017). “Classical organic acidurias”: diagnosis and pathogenesis. Clin Exp Med.

[9] Chapman KA, Gropman A, MacLeod E, Stagni K, Summar ML, Ueda K (2012). Acute management of propionic acidemia. Mol Genet Metab.

[10] Ogier De Baulny H, Saudubray JM (2002). Branched-chain organic acidurias. Semin Neonatol.

[11] Gilbert-Barness E, Farrell PM (2016). Approach to diagnosis of metabolic diseases. Transl Sci Rare Dis.

[12] Fraser JL, Venditti CP (2016). Methylmalonic and propionic acidemias: clinical management update. Curr Opin Pediatr.

[13] Filipowicz HR, Ernst SL, Ashurst CL, Pasquali M, Longo N (2006). Metabolic changes associated with hyperammonemia in patients with propionic acidemia. Mol Genet Metab.

[14] Rice GM, Steiner RD (2016). Inborn errors of metabolism (metabolic disorders). Pediatr Rev.

[15] Nyhan WL, Kölker S, Hoffmann GF, Hoffmann GF, Nyhan WL, Zschocke J (2017). Metabolic emergencies. Inherited metabolic diseases. A clinical approach.

[16] Thomas JA, Bernstein LE, Helm JR, Rohr F (2015). Organic acidemias. Nutrition Management of Inherited Metabolic Disease: lessons from Metabolic University.

[17] Schiff M, de Baulny HO, Dionisi-Vici C, Saudubray J-M, Baumgartner MR, Walter J (2016). Branched-chain organic acidurias /acidaemias. Inborn metabolic diseases. Diagnosis and treatment.

[18] Chakrapani A, Cleary MA, Wraith JE (2001). Detection of inborn errors of metabolism in the newborn. Arch Dis Child Fetal Neonatal Ed.

[19] Nyhan WL, Bay C, Beyer EW, Mazi M (1999). Neurologic nonmetabolic presentation of propionic acidemia. Arch Neurol.

[20] Parisi E, Nicotera A, Alagna A, Di Rosa G (2015). Neonatal seizures and inborn errors of metabolism: an update. Int J Pediatr Neonatal Care.

[21] Deodato F, Boenzi S, Santorelli FM, Dionisi-Vici C (2006). Methylmalonic and propionic aciduria. Am J Med Genet C Semin Med Genet.

[22] Sag E, Cebi AH, Kaya G, Karaguzel G, Cakir M, Bonham J (2017). A rare cause of recurrent acute pancreatitis in a child: Isovaleric acidemia with novel mutation. Pediatr Gastroenterol Hepatol Nutr.

[23] Gilbert-Barness E, Barness LA (1999). Isovaleric acidemia with promyelocytic myeloproliferative syndrome. Pediatr Dev Pathol.

[24] Budd M, Tanaka K, Holmes L, Efron M, Crawford J, K I (1967). Isovaleric acidaemia. N Engl J Med.

[25] De Keyzer Y, Valayannopoulos V, Benoist JF, Batteux F, Lacaille F, Hubert L (2009). Multiple OXPHOS deficiency in the liver, kidney, heart, and skeletal muscle of patients with methylmalonic aciduria and propionic aciduria. Pediatr Res.

[26] Prunty H, Hollak CEMLR (2016). Branched chain amino acids. Inherited metabolic disease in adults. A clinical guide.

[27] Ledley FD, Rosenblatt DS (1997). Mutations in Mut methylmalonic acidemia: clinical and enzymatic correlations. Hum Mutat.

[28] Dobson CM, Gradinger A, Longo N, Wu X, Leclerc D, Lerner-Ellis J (2006). Homozygous nonsense mutation in the MCEE gene and siRNA suppression of methylmalonyl-CoA epimerase expression: a novel cause of mild methylmalonic aciduria. Mol Genet Metab.

[29] Vianey-Saban C, Acquaviva-Bourdain C, Levrat V, Boyer S, Forest I, Fouilhoux A, Bachmann C, Haberle J, Leonard J (2006). Role of N-carbamylglutamate in undiagnosed neonatal hyperammonaemia. Pathophysiology and Management of Hyperammonaemia.

[30] Haussinger D, Lamers WH, Moorman AF (1992). Hepatocyte heterogeneity in the metabolism of amino acids and ammonia. Enzyme.

[31] Dercksen M, IJlst L, Duran M, Mienie LJ, van Cruchten A, van der Westhuizen FH (2014). Inhibition of N-acetylglutamate synthase by various monocarboxylic and dicarboxylic short-chain coenzyme a esters and the production of alternative glutamate esters. Biochim Biophys Acta - Mol Basis Dis.

[32] Haberle J, Boddaert N, Burlina A, Chakrapani A, Dixon M, Huemer M (2012). Suggested guidelines for the diagnosis and management of urea cycle disorders. Orphanet J Rare Dis.

[33] Coude FX, Sweetman L, Nyhan WL (1979). Inhibition by propionyl-coenzyme a of N-acetylglutamate synthetase in rat liver mitochondria. J Clin Invest.

[34] Stewart PM, Walser M (1980). Failure of the normal ureagenic response to amino acids in organic acid-loaded rats. Proposed mechanism for the hyperammonemia of propionic and methylmalonic acidemia. J Clin Invest.

[35] Valayannopoulos V, Baruteau J, Delgado MB, Cano A, Couce ML, Del Toro M (2016). Carglumic acid enhances rapid ammonia detoxification in classical organic acidurias with a favourable risk-benefit profile: a retrospective observational study. Orphanet J Rare Dis.

[36] Braissant O, McLin VA, Cudalbu C (2013). Ammonia toxicity to the brain. J Inherit Metab Dis.

[37] Han L, Wu S, Han F, Gu X (2015). Insights into the molecular mechanisms of methylmalonic acidemia using microarray technology. Int J Clin Exp Med.

[38] Zsengellér ZK, Aljinovic N, Teot LA, Korson M, Rodig N, Sloan JL (2014). Methylmalonic acidemia: a megamitochondrial disorder affecting the kidney. Pediatr Nephrol.

[39] Salmi H, Leonard JV, Lapatto R (2012). Patients with organic acidaemias have an altered thiol status. Acta Paediatr Int J Paediatr.

[40] Jafari P, Braissant O, Zavadakova P, Henry H, Bonafé L, Ballhausen D (2013). Brain damage in methylmalonic aciduria: 2-methylcitrate induces cerebral ammonium accumulation and apoptosis in 3D organotypic brain cell cultures. Orphanet J Rare Dis.

[41] Mitchell GA, Gauthier N, Lesimple A, Wang SP, Mamer O, Qureshi I (2008). Hereditary and acquired diseases of acyl-coenzyme a metabolism. Mol Genet Metab.

[42] Grunert SC, Mullerleile S, De Silva L, Barth M, Walter M, Walter K (2013). Propionic acidemia: clinical course and outcome in 55 pediatric and adolescent patients. Orphanet J Rare Dis.

[43] Nizon M, Ottolenghi C, Valayannopoulos V, Arnoux J-B, Barbier V, Habarou F (2013). Long-term neurological outcome of a cohort of 80 patients with classical organic acidurias. Orphanet J Rare Dis.

[44] Pena L, Franks J, Chapman KA, Gropman A, Ah Mew N, Chakrapani A (2012). Natural history of propionic acidemia. Mol Genet Metab.

[45] Hörster F, Baumgartner MR, Viardot C, Suormala T, Burgard P, Fowler B (2007). Long-term outcome in methylmalonic acidurias is influenced by the underlying defect (mut0, Mut-, cblA, cblB). Pediatr Res.

[46] Kölker S, Valayannopoulos V, Burlina AB, Sykut-Cegielska J, Wijburg FA, Teles EL (2015). The phenotypic spectrum of organic acidurias and urea cycle disorders. Part 2: the evolving clinical phenotype. J Inherit Metab Dis.

[47] Aldubayan S, Rodan L, Berry G, Levy H (2017). Acute illness protocol for organic acidemias: Methylmalonic acidemia and propionic acidemia. Pediatr Emerg Care.

[48] Ito T, Kidouchi K, Sugiyama N, Kajita M, Chiba T, Niwa T (1995). Liquid chromatographic-atmospheric pressure chemical ionization mass spectrometric analysis of glycine conjugates and urinary isovalerylglycine in isovaleric acidaemia. J Chromatogr B.

[49] Kleijer WJ, van der Kraan M, Huijmans JG, van den Heuvel CM, Jakobs C (1995). Prenatal diagnosis of isovaleric acidaemia by enzyme and metabolite assay in the first and second trimesters. Prenat Diagn.

[50] Kölker S, Garcia-Cazorla A, Cazorla Garcia A, Valayannopoulos V, Lund M, Burlina B (2015). The phenotypic spectrum of organic acidurias and urea cycle disorders. Part 1: the initial presentation. J Inherit Metab Dis.

[51] Scholl-Bürgi S, Sass JO, Zschocke J, Karall D (2012). Amino acid metabolism in patients with propionic acidaemia. J Inherit Metab Dis.

[52] Opladen T, Cortez-Saladelafont E, Mastrangelo M, Horvath G, Pons R, Lopez-Laso E (2016). The international working group on neurotransmitter related disorders (iNTD): a worldwide research project focused on primary and secondary neurotransmitter disorders. Mol Genet Metab Reports.

[53] Burton BK (1998). Inborn errors of metabolism in infancy: a guide to diagnosis. Pediatrics.

[54] Elsas LJ, Naglak M (1988). Acute and chronic-intermittent isovaleric acidaemia: diagnosis and glycine therapy. Acta Paediatr Jpn.

[55] Chalmers RA, de Sousa C, Tracey BM, Stacey TE, Weaver C, Bradley D (1985). L-carnitine and glycine therapy in isovaleric acidaemia. J Inherit Metab Dis.

[56] Daniotti M, la Marca G, Fiorini P, Filippi L (2011). New developments in the treatment of hyperammonemia: emerging use of carglumic acid. Int J Gen Med.

[57] Häberle J (2011). Role of carglumic acid in the treatment of acute hyperammonemia due to N-acetylglutamate synthase deficiency. Ther Clin Risk Manag.

[58] Chakrapani A, Valayannopoulos V, Segarra NG, Del Toro M, Donati MA, García-cazorla A (2018). Effect of carglumic acid with or without ammonia scavengers on hyperammonaemia in acute decompensation episodes of organic acidurias. Orphanet J Rare Dis.

[59] Filippi L, Gozzini E, Fiorini P, Malvagia S, La Marca G, Donati MA (2010). N-carbamylglutamate in emergency management of hyperammonemia in neonatal acute onset propionic and methylmalonic aciduria. Neonatology.

[60] Gebhardt B, Dittrich S, Parbel S, Vlaho S, Matsika O, Bohles H (2005). N-Carbamylglutamate protects patients with decompensated propionic aciduria from hyperammonaemia. J Inherit Metab Dis.

[61] Sutton VR, Chapman KA, Gropman AL, MacLeod E, Stagni K, Summar ML (2012). Chronic management and health supervision of individuals with propionic acidemia. Mol Genet Metab.

[62] Pinto A, Daly A, Evans S, Almeida MF, Assoun M, Belanger-Quintana A (2017). Dietary practices in isovaleric acidemia: a European survey. Mol Genet Metab Reports.

[63] Heringer J, Valayannopoulos V, Lund AM, Wijburg FA, Freisinger P, Baric I (2016). Impact of age at onset and newborn screening on outcome in organic acidurias. J Inherit Metab Dis.

[64] Longo N, Price LB, Gappmaier E, Cantor NL, Ernst SL, Bailey C (2017). Anaplerotic therapy in propionic acidemia. Mol Genet Metab.

[65] Berry GT, Yudkoff M, Segal S (1988). Isovaleric acidemia: medical and neurodevelopmental effects of long-term therapy. J Pediatr.

[66] Mayatepek E, Kurczynski TW, Hoppel CL (1991). Long-term L-carnitine treatment in isovaleric acidemia. Pediatr Neurol.

[67] Burlina A, Cazzorla C, Zanonato E, Viggiano E, Fasan I, Polo G (2016). Clinical experience with N-carbamylglutamate in a single-Centre cohort of patients with propionic and methylmalonic aciduria. Mol Genet Metab Reports.

[68] Tummolo A, Melpignano L, Carella A, Di Mauro AM, Piccinno E, Vendemiale M (2018). Long-term continuous N-carbamylglutamate treatment in frequently decompensated propionic acidemia: a case report. J Med Case Rep.

[69] McCrory NM, Edick MJ, Ahmad A, Lipinski S, Scott Schwoerer JA, Zhai S (2017). Comparison of methods of initial ascertainment in 58 cases of propionic acidemia enrolled in the inborn errors of metabolism information system reveals significant differences in time to evaluation and symptoms at presentation. J Pediatr.

